# Aortic valve surgery in adolescents and young adults: analysis of early operative data from the European Congenital Heart Surgeons Association database^†^

**DOI:** 10.1093/ejcts/ezaf101

**Published:** 2025-06-26

**Authors:** Alvise Guariento, Claudia Cattapan, Ilias P Doulamis, Zdzislaw Tobota, Bohdan Maruszewski, Mark S Bleiweis, Jeffrey P Jacobs, George E Sarris, Vladimiro Vida

**Affiliations:** Pediatric and Congenital Cardiac Surgery Unit, Department of Cardiac, Thoracic, Vascular Sciences and Public Health, University of Padua, Padua, Italy; Pediatric and Congenital Cardiac Surgery Unit, Department of Cardiac, Thoracic, Vascular Sciences and Public Health, University of Padua, Padua, Italy; Department of Surgery, Lahey Clinic, Tufts University School of Medicine, Burlington, MA, USA; Pediatric Cardiothoracic Surgery, Children’s Memorial Health Institute, Warsaw, Poland; Pediatric Cardiothoracic Surgery, Children’s Memorial Health Institute, Warsaw, Poland; Congenital Heart Center, Division of Cardiovascular Surgery, Departments of Surgery and Pediatrics, University of Florida, Gainesville, FL, USA; Congenital Heart Center, Division of Cardiovascular Surgery, Departments of Surgery and Pediatrics, University of Florida, Gainesville, FL, USA; Athens Heart Surgery Institute, Department of Pediatric Heart Surgery, Mitera Children’s Hospital, Athens, Greece; ECHSA Congenital Database Committee Chair, Department of Pediatric Heart Surgery, Mitera Children’s Hospital, Athens, Greece; Pediatric and Congenital Cardiac Surgery Unit, Department of Cardiac, Thoracic, Vascular Sciences and Public Health, University of Padua, Padua, Italy

**Keywords:** Aortic valve surgery, Adolescents, Young adults, European Association of Congenital Heart Surgeons, Database

## Abstract

**OBJECTIVES:**

Aortic valve surgery is a crucial treatment for congenital and acquired aortic disease in adolescents and young adults. This study evaluated outcomes in this group by analysing data from the European Congenital Heart Surgeons Association Congenital Cardiac Database (ECCDB).

**METHODS:**

A retrospective review included patients aged 10–18 years from the ECCDB who underwent aortic valve surgery between 2013 and 2022. The primary outcome was operative mortality, defined as death within 30 days or during hospitalization. Secondary outcomes included reoperations and postoperative complications. Risk factors for mortality were identified using multivariable logistic regression analysis, and surgical trends were evaluated.

**RESULTS:**

A total of 2129 patients were included, with the majority undergoing valve replacement, followed by valve repair and the Ross procedure. Patients receiving valve replacement were typically older and larger. Over the decade, there was an increase in the use of the Ross procedure. Reoperations were more frequent in the repair group, while postoperative complications were more common in the replacement group. The overall mortality rate was 1.5%. Independent risk factors for mortality included longer cardiopulmonary bypass (CPB) times (odds ratio 1.1, *P* < 0.001) and annulus enlargement (odds ratio 3.8, *P* = 0.02). CPB durations exceeding 240 min increased the risk of death. The Ross procedure, particularly in isolated cases without annulus enlargement, was associated with a low mortality rate of 0.4%.

**CONCLUSIONS:**

Aortic valve surgery in adolescents and young adults is complex, with outcomes influenced by CPB time and annulus enlargement. The Ross procedure shows excellent results despite its technical demands.

## GRAPHICAL ABSTRACT

**Figure:**
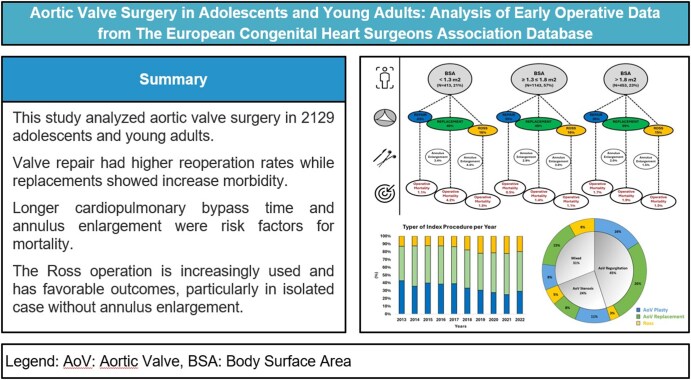

## INTRODUCTION

Aortic valve surgery, including both repair and replacement, is a foundation of the management of congenital and acquired aortic valve disorders in adolescents and young adults [1]. This population presents unique challenges in surgical management due to continued growth, potential for long-term survival and diverse aetiologies of aortic valve disease or other associated congenital heart disease (CHD) [2].

Selecting an appropriate surgical approach for this demographic requires careful consideration of multiple factors, including the patient’s age, sex, body size, activity level and specific characteristics of the valve disease [3–5]. Additionally, considerations regarding the potential for future surgical interventions, longevity of the valve prosthesis and management of anticoagulation therapy, particularly in female patients with potential pregnancies, play a critical role in the decision-making process [4]. Despite advances in surgical techniques and technology, significant challenges remain in optimizing outcomes for adolescents and young adults undergoing aortic valve surgery.

The aim of this study was to provide a comprehensive review of the status of aortic valve surgery in adolescents and young adults over a span of 10 years, drawing on data from the European Association of Congenital Heart Surgeons (ECHSA) Congenital Cardiac database (ECCDB).

## PATIENTS AND METHODS

### Study design

A multicentre retrospective analysis was conducted using data from the European Congenital Heart Surgeons Association Congenital Cardiac Database (ECCDB) [6]. The study included patients aged 10–18 years who underwent aortic valve surgery between 2013 and 2022 (Supplementary Material, Methods) [7, 8]. Exclusion criteria comprised patients who underwent interventional cardiology procedures, those who had ascending aortic surgery without concurrent aortic valve surgery, duplicate medical records or cases of multiple entries during the same study period but with separate hospitalizations, considering that a complete follow-up for all patients after discharge was not available. Procedures recorded separately but performed on the same patient within the same hospitalization were identified and included as reoperations (Supplementary Material, Fig. S1).

### Data source

The characteristics and data verification processes of the ECCDB have been previously documented [9, 10]. Data are periodically verified for completeness and accuracy by trained personnel [9]. The data, provided by the ECCDB after study review and approval by the ECCDB Committee for compliance with all ECHSA ethics and patient data protection policies, validated in accordance with ECCDB procedures, include fully anonymized information on patients undergoing congenital cardiac surgery in participating hospitals. For this reason, patient consent was waived.

### Outcomes

The primary end-point of the study was operative mortality. Secondary outcome included reoperations during the same hospitalization (regardless of discharge timing) and postoperative complications. Operative mortality was defined as 30-day mortality or in-hospital mortality, based on the dates of death, surgery and discharge. Specifically, 30-day mortality refers to any death occurring within 30 days of surgery, regardless of cause or location, while in-hospital mortality refers to any death occurring during the same hospital stay as cardiac surgery, regardless of cause or time since surgery.

### Statistical analysis

Categorical data are presented as frequencies and/or percentages. Continuous variables are presented as medians and interquartile ranges (Tables 1–3, Supplementary Material, Tables S1–S3). Group comparisons for continuous variables were conducted using a one-way measures analysis of variance, while the chi-square test was applied for categorical variables (Tables 1–3).

**Table 1:** Patient demographics and preoperative data (*N*, % or median, IQR)

	Overall	Repair	Replacement	Ross	*P*-value
	(*N* = 2129)	(*N* = 742, 35%)	(*N* = 1044, 49%)	(*N* = 343, 16%)	
Demographics					
Age at index surgery (years)	14 (12, 16)	14 (12, 16)	15 (13, 17)	14 (12, 16)	**<0.001**
Male gender	1570 (74%)	535 (72%)	806 (77%)	229 (67%)	**<0.001**
Weight (kg)	53 (42, 66)	50 (40, 61)	56 (44, 69)	51 (42, 64)	**<0.001**
Height (cm)	162 (151, 173)	159 (148, 169)	166 (154, 175)	162 (150, 172)	**<0.001**
BSA (m^2^)	1.6 (1.3, 1.8)	1.5 (1.3, 1.7)	1.6 (1.4, 1.8)	1.5 (1.3, 1.8)	**<0.001**
Associated disease					
Associated CHD at initial diagnosis	420 (20%)	155 (21%)	226 (22%)	39 (11%)	**<0.001**
VSD (any form)	120 (5.6%)	59 (8.0%)	48 (4.6%)	8 (2.3%)	**<0.001**
D-TGA	61 (2.9%)	16 (2.2%)	45 (4.3%)	0 (0%)	**<0.001**
Isolated aortic coarctation	53 (2.5%)	14 (1.9%)	22 (2.1%)	12 (3.5%)	0.22
Truncus arteriosus	40 (1.9%)	14 (1.9%)	26 (2.5%)	0 (0%)	**0.014**
AVSD (complete or partial)	24 (1.1%)	9 (1.2%)	11 (1.1%)	4 (1.2%)	0.9
CHD requiring RV-PA conduit	19 (0.9%)	1 (0.1%)	18 (1.7%)	0 (0%)	**<0.001**
DORV (any form)	19 (0.9%)	7 (0.9%)	11 (1.1%)	1 (0.3%)	0.42
TOF/TOF-PA	18 (0.8%)	8 (1.1%)	10 (1.0%)	0 (0%)	0.17
Univentricular physiology	16 (0.8%)	3 (0.4%)	12 (1.1%)	1 (0.3%)	0.11
AAOCA/coronary fistula	14 (0.7%)	11 (1.5%)	3 (0.3%)	0 (0%)	**0.002**
Aortic arch hypoplasia	11 (0.5%)	2 (0.3%)	7 (0.7%)	2 (0.6%)	0.50
Interrupted aortic arch	11 (0.5%)	4 (0.5%)	4 (0.4%)	3 (0.9%)	0.54
Shone’s complex	10 (0.5%)	4 (0.5%)	5 (0.5%)	1 (0.3%)	0.86
Other minor CHD	16 (0.8%)	3 (0.4%)	10 (1.0%)	3 (0.9%)	0.39
Associated genetic disease	102 (4.8%)	38 (5.1%)	55 (5.3%)	9 (2.6%)	0.12
Marfan syndrome	32 (1.5%)	11 (1.5%)	21 (2.0%)	0 (0%)	**0.032**
Other syndromes	70 (3.3%)	27 (3.6%)	34 (3.2%)	9 (2.6%)	0.24
Preoperative data					
Mechanism of aortic valve disease					**<0.001**
Aortic valve regurgitation	948 (45%)	343 (46%)	552 (53%)	53 (15%)	
Aortic valve stenosis	520 (24%)	231 (31%)	174 (17%)	115 (34%)	
Mixed aortic valve disease	661 (31%)	168 (23%)	318 (30%)	175 (51%)	
Endocarditis on the aortic valve	84 (3.9%)	21 (2.8%)	56 (5.4%)	7 (2.0%)	**0.004**
Rheumatic aortic valve disease	164 (8.2%)	50 (6.7%)	116 (11%)	3 (0.9%)	**<0.001**
Aortic aneurysm	238 (11.2%)	54 (7.3%)	158 (15%)	26 (7.6%)	**<0.001**
Aortic dissection	6 (0.3%)	2 (0.3%)	4 (0.4%)	0 (0%)	0.50

AAOCA: anomalous aortic origin of the coronary artery; AVSD: atrioventricular septal defect; CHD: congenital heart disease; D-TGA: D-transposition of the great arteries; DORV: double outlet right ventricle; IQR: interquartile range; RV-PA: right ventricular-to-pulmonary artery; TOF/PA: tetralogy of Fallot/pulmonary artery; VSD: ventricular septal defect.

In bold: significant p-values (<0.05).

**Table 2:** Operative data (*N*, % or median, IQR)

	Overall	Repair	Replacement	Ross	*P*-value
	(*N* = 2129)	(*N* = 742, 35%)	(*N* = 1044, 49%)	(*N* = 343, 16%)	
Index procedure					
Period of surgery					**<0.001**
2013–2017	1199 (56%)	471 (63%)	576 (55%)	152 (44%)	
2018–2022	930 (44%)	271 (37%)	468 (45%)	191 (56%)	
Redo surgery	319 (15%)	93 (13%)	194 (19%)	34 (9.9%)	**<0.001**
Failed initial index procedure	47 (2.2%)	38 (5.1%)	4 (0.4%)	5 (1.5%)	**<0.001**
Type of index valve replacement					
Bioprosthetic			227 (22%)		
Mechanical			748 (72%)		
NOS			65 (6.2%)		
Bentall procedure			291 (28%)		
Aortic root replacement W/repair		18 (2.4%)			
Annulus enlargement	130 (6.1%)	4 (0.5%)	59 (5.7%)	67 (20%)	**<0.001**
Konno enlargement	102 (4.8%)	4 (0.5%)	33 (3.2%)	65 (19%)	**<0.001**
Other type of enlargement	28 (1.3%)	0 (0%)	26 (2.5%	2 (0.6%)	**<0.001**
Associated procedure					
Associated cardiac procedure	757 (36%)	335 (45%)	361 (35%)	61 (18%)	**<0.001**
Mitral valve repair/replacement	238 (11%)	80 (11%)	148 (14%)	10 (2.9%)	**<0.001**
Subaortic stenosis repair	188 (8.8%)	133 (18%)	49 (4.7%)	6 (1.7%)	**<0.001**
Procedures on RV-PA Conduit/RVOT	121 (5.7%)	28 (3.8%)	63 (6.0%)	–	**0.004**
Aortic aneurysm repair	102 (4.8%)	36 (4.9%)	52 (5.0%)	14 (4.1%)	0.79
VSD closure	83 (3.9%)	49 (6.6%)	32 (3.1%)	2 (0.6%)	**<0.001**
Tricuspid valve repair/replacement	56 (2.6%)	21 (2.8%)	34 (3.3%)	1 (0.3%)	**0.012**
Procedures on pulmonary arteries	45 (2.1%)	11 (1.5%)	34 (3.3%)	0 (0%)	**<0.001**
Supraortic stenosis repair	43 (2.0%)	30 (4.0%)	13 (1.2%)	0 (0%)	**<0.001**
Pulmonary valve repair/replacement	34 (1.6%)	8 (1.1%)	24 (2.3%)	–	**0.033**
Procedure on coronary arteries	22 (1.0%)	10 (1.3%)	11 (1.1%)	1 (0.3%)	0.3
Procedure on other minor CHD	15 (0.7%)	3 (0.4%)	13 (1.2%)	1 (0.3%)	**0.012**
Intraoperative data					
Minimally invasive access	13 (0.6%)	3 (0.04%)	8 (0.8%)	2 (0.6%)	0.62
CPB time (min)	144 (102, 204)	114 (79, 156)	150 (109, 210)	205 (165, 259)	**<0.001**
AOX time (min)	102 (69, 145)	76 (48, 113)	103 (75, 145)	147 (120, 178)	**<0.001**
Circulatory arrest	37 (1.7%)	11 (1.5%)	26 (2.5%)	0 (0%)	**0.007**
Circulatory arrest time (min)	20 (7, 32)	32 (14, 35)	19 (7, 25)	–	0.23

AOX: aortic cross-clamp; CHD: congenital heart disease; CPB: cardiopulmonary bypass; IQR: interquartile range; NOS: not otherwise specified; RV-PA: right ventricular-to-pulmonary artery; VSD: ventricular septal defect.

In bold: significant p-values (<0.05).

**Table 3:** Postoperative data (*N*, % or median, IQR)

	Overall	Repair	Replacement	Ross	*P*-value
	(*N* = 2129)	(*N* = 742, 35%)	(*N* = 1044, 49%)	(*N* = 343, 16%)	
Hospitalization					
POD at ICU discharge	2 (1, 3)	2 (1, 3)	2 (1, 4)	2 (2, 4)	**<0.001**
POD at final discharge	8 (6, 13)	7 (6, 10)	10 (7, 15)	8 (7, 11)	**<0.001**
Reoperation within same hospitalization					
Reoperation on the aortic valve	34 (1.6%)	25 (3.4%)	8 (0.8%)	1 (0.3%)	**<0.001**
Type of reoperation					
Aortic valve repair	9 (1.2%)	7 (0.9%)	0 (0%)	0 (0%)	
Aortic valve replacement	21 (1.0%)	14 (1.9%)	8 (0.8%)	1 (0.3%)	
Ross procedure	4 (0.2%)	4 (0.5%)	0 (0%)	0 (0%)	
Postoperative complication					
Major complication requiring treatment	319 (15%)	72 (9.7%)	198 (19%)	49 (14%)	**<0.001**
Arrhythmia requiring drugs or TPM	86 (4.0%)	21 (2.8%)	52 (5.0%)	13 (3.8%)	0.073
Bleeding requiring reoperation	73 (3.4%)	22 (3.0%)	41 (3.9%)	10 (2.9%)	0.46
Pleural effusion/pneumothorax	51 (2.4%)	10 (1.3%)	32 (3.1%)	9 (2.6%)	0.062
Pericardial effusion	49 (2.3%)	12 (1.6%)	31 (3.0%)	6 (1.7%)	0.13
LCOS	39 (1.8%)	7 (0.9%)	21 (2.0%)	11 (3.2%)	**0.029**
Arrhythmia requiring PPM	32 (1.5%)	7 (0.9%)	23 (2.2%)	2 (0.6%)	**0.030**
Respiratory failure/pneumonia	26 (1.2%)	3 (0.4%)	18 (1.7%)	5 (1.5%)	**0.040**
ECMO	23 (1.1%)	4 (0.05%)	14 (1.3%)	5 (1.5%)	0.21
Major infection/sepsis	17 (0.8%)	3 (0.4%)	12 (1.1%)	2 (0.6%)	0.19
Renal failure	17 (0.8%)	1 (0.1%)	13 (1.2%)	3 (0.9%)	**0.034**
Wound infection	15 (0.7%)	1 (0.1%)	11 (1.1%)	3 (0.9%)	0.06
Neurological injury	9 (0.4%)	0 (0%)	6 (0.6%)	3 (0.9%)	0.068
Endocarditis	4 (0.2%)	0 (0%)	4 (0.4%)	0 (0%)	**0.004**
Mortality					
Operative mortality	31 (1.5%)	6 (0.8%)	20 (1.9%)	5 (1.5%)	0.25
Mortality w/o annulus enlargement	25 (1.3%)	6 (0.8%)	18 (1.8%)	1 (0.4%)	0.062
POD at death	14 (3, 22)	7 (4, 12)	15 (2, 22)	18 (14, 23)	0.27

ECMO: extracorporeal membrane oxygenation; IQR: interquartile range; LCOS: low cardiac output syndrome; POD: postoperative day; PPM: permanent pacemakeer; TPM: temporary pacemaker.

In bold: significant p-values (<0.05).

Univariate and multivariable logistic regression models were used to identify factors associated with operative mortality and reoperation within the same hospitalization, with results reported as odds ratios (ORs) and 95% confidence intervals (not adjusted for multiple comparisons). For the multivariable analysis, covariates included in the model were selected based on their potential clinical relevance and the findings from group analyses (Tables 1–3, Supplementary Material, Tables S1–S4). These were: age at surgery, gender, weight, body surface area (BSA), associated CHD at initial diagnosis, associated genetic disease, mechanism of aortic valve disease, period of surgery, redo surgery (aortic valve procedure in a previous admission), failed initial index procedure (during the same admission), type of index procedure, annulus enlargement, length of cardiopulmonary bypass (CPB) and aortic cross-clamp (AOX) as continuous variables, other associated cardiac procedures concomitant to the index procedure. Receiver operating characteristic curves were plotted to identify the optimal CPB threshold for predicting mortality (Supplementary Material, Fig. S2). Statistical significance was defined as a two-sided alpha of 0.05. All analyses were performed using Stata 17 (StataCorp LLC, College Station, TX, USA).

## RESULTS

### Demographics, operative data and postoperative outcomes in the entire population and by type of index procedure

**Figure 1:**
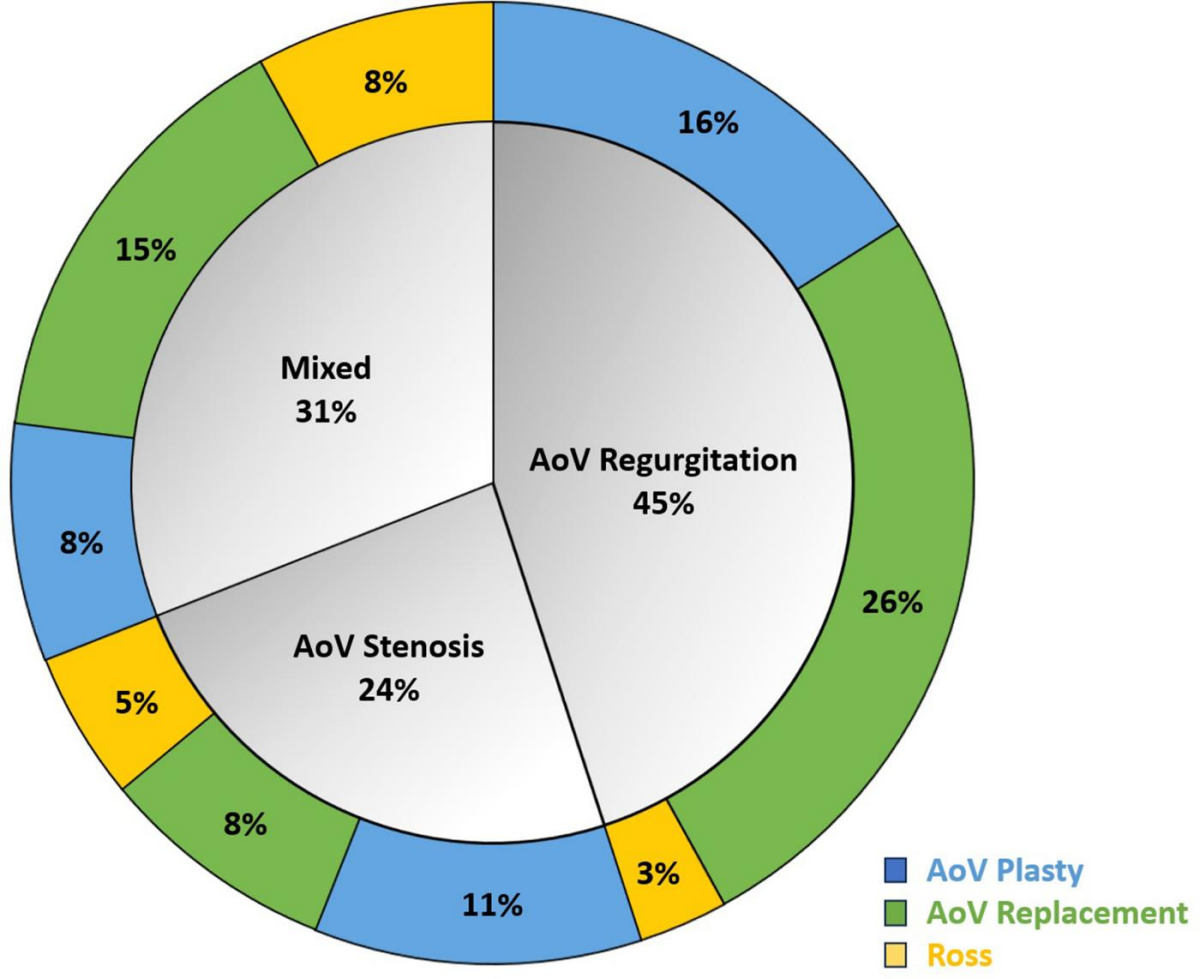
Proportions of patients by mechanism of aortic valve disease and type of treatment. AoV: aortic valve.

In this study, 2129 patients from 34 centres were analysed from an initial pool of 2264, with 135 exclusions. Of these, 35% underwent surgical repair, 49% valve replacement and 16% the Ross procedure (Tables 1–3 and Fig. 1).

**Figure 2:**
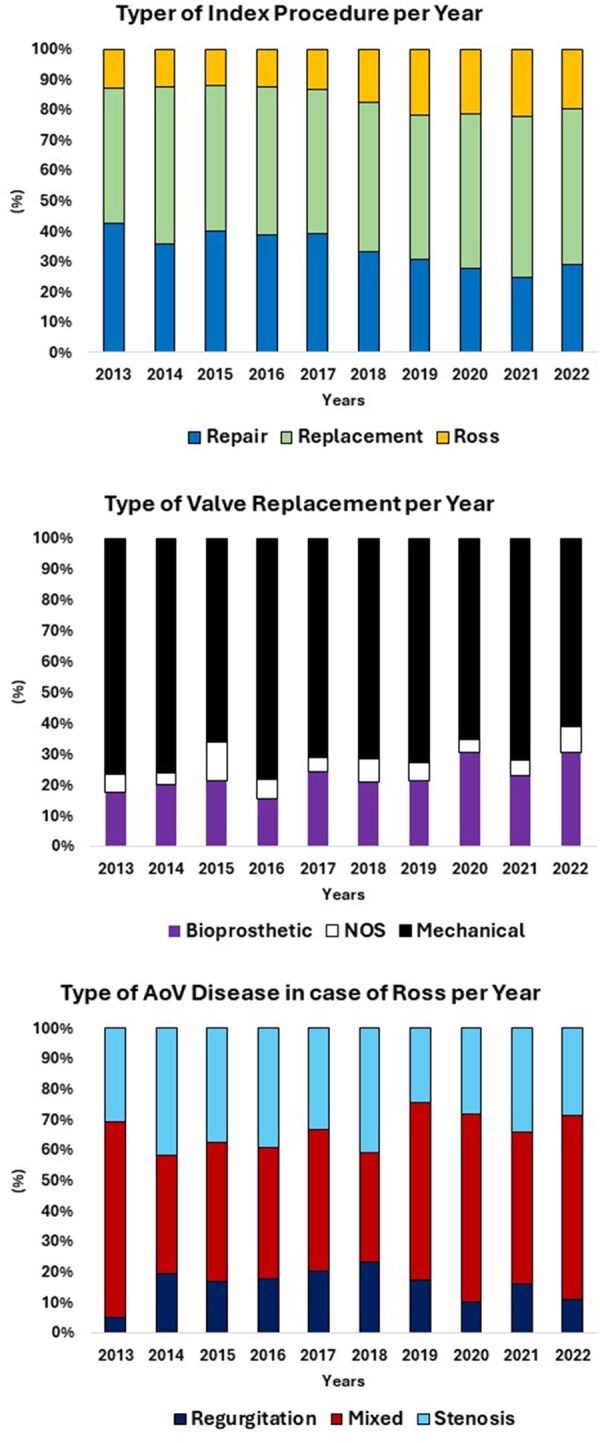
Population trends for different groups over the years.

Preoperative data showed that patients who underwent valve replacement were older than the repair and Ross groups (*P* < 0.001) (Table 1). The replacement group had a higher proportion of males (77%) and a higher median weight and height than the repair and Ross groups (*P* < 0.001). The same was observed when looking at BSA, which was significantly lower in the repair and Ross groups compared to the replacement group (*P* < 0.001). The Ross group had a lower incidence of associated CHD (*P* < 0.001). The overall number of associated genetic diseases did not differ between the groups, but Marfan disease was more common in the replacement group (*P* = 0.03). Preoperative data indicated that aortic valve regurgitation was more common in the replacement group (53%), while aortic stenosis was more common in the Ross group (34%). Mixed aortic valve disease was significantly higher in the Ross group (51%) (*P* < 0.001) and this did not change over the years (Figs 1 and 2). Aortic aneurysms (15%) (*P* < 0.001) and endocarditis (5.4%) (*P* = 0.004) were more common in the replacement group, as was rheumatic aortic valve disease (11%) (*P* < 0.001).

Fifty-six percent of surgeries occurred between 2013 and 2017, while 44% occurred in the second decade between 2018 and 2022. There was a decrease in procedures in both the repair and replacement groups and an increase in the Ross group (*P* < 0.001) (Table 2 and Fig. 2). Redo surgeries were more common in the replacement group (19%) (*P* < 0.001). The incidence of failed initial procedures was highest in the repair group (5.1%) (*P* < 0.001).

Of the valve replacements, 72% received mechanical valves, 22% bioprostheses and 6.2% were unspecified. The type of prosthetic valve used over the years has not changed significantly (Fig. 2). The Bentall procedure was performed in 28% of patients undergoing replacement, while aortic root replacement with aortic valve repair (David procedure) occurred in 2.4% of patients undergoing repair. Annulus enlargement and Konno enlargement were more common in the Ross group (20% and 19%, respectively) (*P* < 0.001). Associated cardiac procedures were performed in 36% of patients, with the repair group having the highest frequency (45%) and the Ross group the lowest (18%) (*P* < 0.001).

Intraoperative data showed negligible use of minimally invasive access (<1%) in all the 3 groups. CPB times were longest for the Ross procedure, followed by replacement and repair (*P* < 0.001). AOX times were also longer for the Ross group (*P* < 0.001). Circulatory arrest was more common in the replacement group (2.5%) and absent in the Ross group (*P* < 0.001).

Discharge from the intensive care unit was slightly later in Ross patients (*P* < 0.001) (Table 3). Final hospital discharge was earlier for repair patients compared with replacement and Ross patients (*P* < 0.001). Aortic valve reoperations within the same hospital stay were more common in the repair group (3.4%) (*P* < 0.001). Postoperative complications were higher in the replacement group (*P* < 0.001).

Overall operative mortality was 1.5% and was slightly higher in the replacement group (1.9%) than repair (0.8%) and Ross (1.5%) but not statistically significant. Mortality without annulus enlargement was also higher in the replacement group (1.8%) and lower in the Ross group (0.4%).

### Risk factors for mortality

Univariate analysis for mortality predictors showed that associated CHD at initial diagnosis significantly increased the risk of mortality (OR 4.5, *P* < 0.001), as did reoperation (OR 7.2, *P* < 0.001), annulus enlargement (OR 3.8, *P* = 0.004), longer CPB time (OR 1.1, *P* < 0.001), AOX time (OR 1.1, *P* = 0.008) and circulatory arrest (OR 6.5, *P* = 0.003) (Supplementary Material, Table S1). The mechanism of aortic valve disease was also significant (OR 0.6, *P* = 0.02). In multivariate analysis, annulus enlargement (OR 3.8, *P* = 0.02) and CPB time (OR 1.1, *P* < 0.001) remained significant predictors of mortality. Other factors, such as reintervention (OR 14.8, *P* = 0.13), associated CHD (OR 0.2, *P* = 0.30) and AOX time (OR 0.9, *P* = 0.06), were not significant after adjustment for other variables (Table 4).

**Table 4:** Predictors for mortality

Multivariable analysis		
Variable	Adjusted OR (95% CI)	*P*-value
Associated CHD at initial diagnosis	0.2 (0.1, 5.1)	0.30
Mechanism of aortic valve disease	0.6 (0.3, 1.1)	0.12
Redo surgery	14.8 (0.5, 475)	0.13
Annulus enlargement	3.8 (1.2, 12)	**0.020**
CPB time	1.1 (1.1, 1.2)	**<0.001**
AOX time	0.9 (0.9, 1.0)	0.06
Circulatory arrest	0.7 (0.1, 0.2)	0.79

Variables with *P* < 0.05 on univariable analysis were included in the multivariable model.

AOX: aortic cross-clamp; CHD: congenital heart disease; CPB: cardiopulmonary bypass.

In bold: significant p-values (<0.05).

### Receiver operating characteristic curve analysis

Sensitivity and specificity analyses were conducted on the significant quantitative variable of the multivariable analysis, CPB time. A CPB time ≥240 min was found to be significant for an increased risk for operative mortality (area under the curve 0.78, sensitivity 62%, specificity 85%). Demographic, intraoperative and postoperative data were then analysed comparing two groups, according to the CPB time (<240 min vs ≥240 min) (Supplementary Material, Table S2).

### Data by other variables

Data were also analysed by the mechanism of aortic valve disease and BSA (Supplementary Material, Fig. 3).

**Figure 3:**
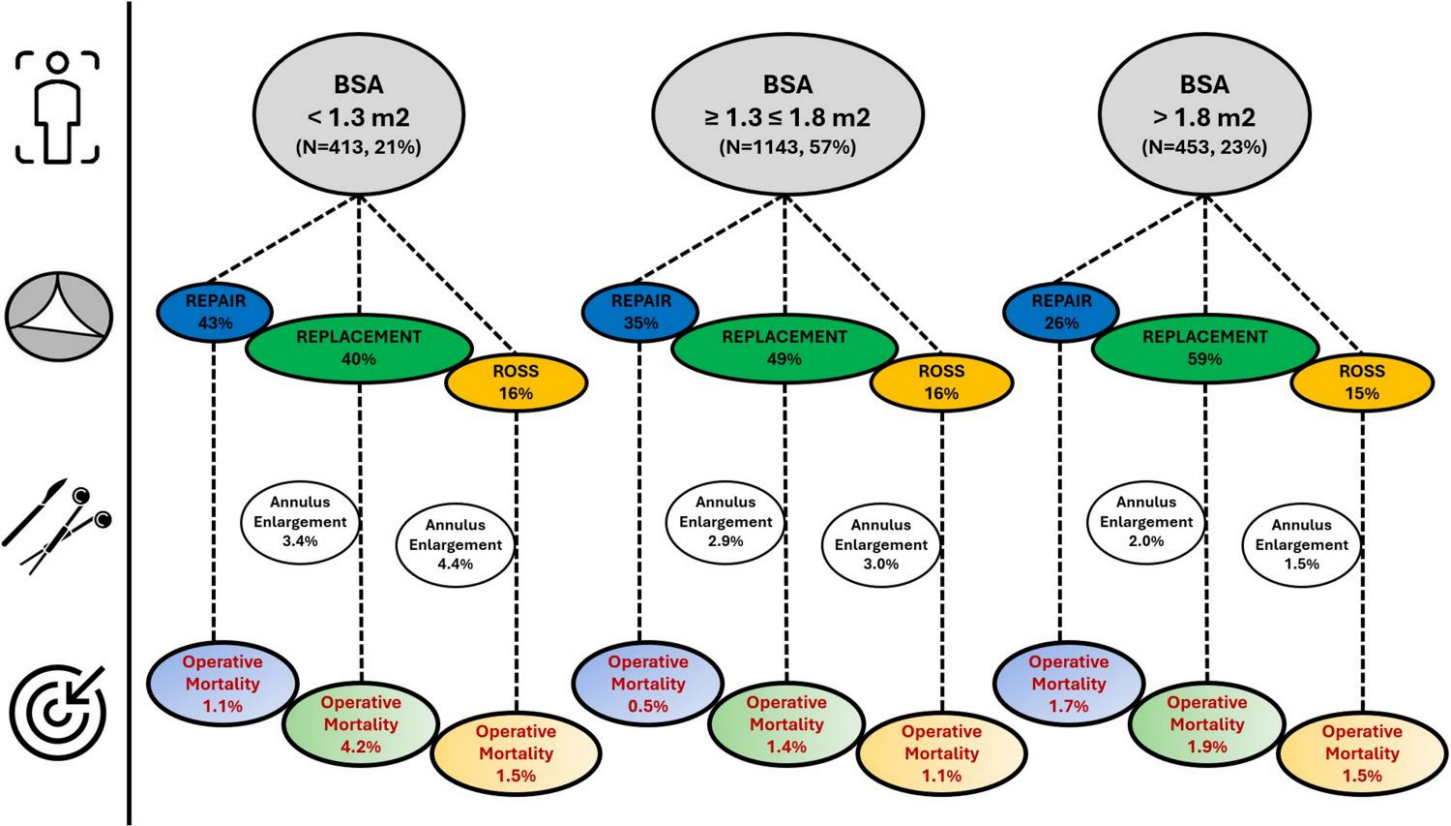
Mortality by body surface area and type of index aortic valve procedure (*N* = 2009). BSA: body surface area.

## DISCUSSION

The management of congenital aortic valve disease in adolescents and young adults is evolving, requiring ongoing research and clinical advances [4, 5, 11]. This study analysed 2129 cases from the ECCDB involving various aortic valve surgeries.

The data confirmed the established link between smaller body size, increased surgical complexity and higher risks [3]. Consistent with previous research, valve replacement was more common among older and larger patients. On the contrary, younger and smaller individuals were more likely to undergo valve repair. However, this choice was associated with higher reoperation rates, as observed in previous studies [12, 13]. Our study also demonstrated low mortality for the Ross procedure in selected patients, consistent with the literature strongly supporting the preference for the Ross procedure in younger patients with aortic stenosis due to excellent early results and its superior long-term haemodynamic outcomes and avoidance of anticoagulation [14, 15]. In contrast, larger patients with associated CHD underwent valve replacement, likely to further increase the complexity of their procedure [3, 16]. This is a crucial point for preoperative counselling and planning, suggesting that surgical teams need to be particularly cautious and perhaps more innovative when managing smaller patients.

The study also documented a shift to the Ross procedure between 2013 and 2022, consistent with broader trends favouring it for younger patients, despite its technical complexity and longer operating times [13, 17, 18]. As supported by other studies, the Ross procedure resulted in longer CPB and AOX times, both of which are associated with increased postoperative risks. Specifically, CPB times greater than 240 min were linked to significantly higher mortality, in line with previous findings that prolonged CPB is associated with worse outcomes [19]. However, the Ross operation did not result in an increased risk of operative mortality in the current study. The optimal timing for the Ross operation remains debated. A recent expert review highlights that this procedure can yield favourable outcomes in infants and children when performed in specialized centres, with autograft stabilization being critical for long-term success in older children, justifying the advocation for Renaissance of the operation [20].

The study reported a low overall mortality rate of 1.5%, consistent with modern surgical outcomes [4]. However, the slightly higher mortality in valve replacement patients highlights the challenges for older patients with advanced diseases. The Ross procedure showed excellent early results, with a mortality rate as low as 0.4% in isolated cases without annulus enlargement. This suggests that the Ross procedure, especially for younger patients, is an effective option and emphasizes the need for personalized surgical strategies.

### Limitations

The main limitation of this study is its retrospective design, which may introduce selection bias and limit causal conclusions. While the cohort is sizable, it comes from a voluntary multicentre database (ECCCDB) with no medium- or long-term follow-up, limiting its generalizability (no out-of-hospital data were included). Future studies should focus on long-term outcomes of different surgical approaches for paediatric and adolescent aortic valve disease.

## CONCLUSIONS

In conclusion, this study highlights the complexity of aortic valve surgery in adolescents and young adults. Valve replacement is more common in older and larger patients, while younger and smaller individuals often undergo valve repair or the Ross procedure. Longer CPB times and annular enlargement are associated with higher mortality. Despite its complexity, the Ross procedure shows excellent outcomes and growing popularity.

## Supplementary Material

ezaf101_Supplementary_Data

## Data Availability

Data supporting the findings of this study are available upon request from the corresponding author. The data are not publicly available due to restrictions imposed because the information may compromise the privacy of research participants.

## ABBREVIATIONS

AOX: Aortic cross-clamp
BSA: Body surface area
CHD: Congenital heart disease
CPB: Cardiopulmonary bypass
ECCDB: European Congenital Heart Surgeons Association Congenital Cardiac Database
ECHSA: European Association of Congenital Heart Surgeons
OR: Odds ratio
