# Supplementary material for: Aortic valve surgery in adolescents and young adults: analysis of early operative data from the European Congenital Heart Surgeons Association database
Source: Eur J Cardiothorac Surg. 2025 Jun 26;67(6):ezaf101. doi: 10.1093/ejcts/ezaf101 (PMC12208064; doi:10.1093/ejcts/ezaf101)
Supplement: ezaf101_Supplementary_Data [file ezaf101_supplementary_data.zip › CORRECT Supplemental Material.docx]

**SUPPLEMENTAL MATERIAL**

**METHODS**

**Study Design**

The mean body surface area for children aged 9 and 10 years is 1.12 m2 and 1.24 m2, respectively, corresponding to mean aortic annulus diameters of 1.63 cm and 1.72 cm. Considering that one of the smallest dedicated aortic valve prostheses (Carbomedics Standard Small Size 16) has an internal diameter of 1.47 cm, we speculated that its use would present an increased intrinsic risk for patients under 10 years of age. This is further supported by a recent meta-analysis of 67 studies involving over 5,000 patients, which investigated pediatric aortic valve replacement. The study reported a mean age for the procedure of 13.0 ± 3.4 years, reinforcing 10 years is the lower feasible age for aortic valve replacement in common clinical practice.

**Data Source**

The ECCCDB is fully compliant with the European General Data Protection Regulation and all participating hospitals have agreed to provide fully anonymized data to the ECDB for use in data analyses for research and quality improvement initiatives in patient care, in full compliance with any applicable local laws and internal Institutional Review Board procedures.

**Statistical Analysis**

The type of Ross procedure was not available in the initial dataset and therefore information regarding potential external reinforcement was not included in the analysis performed.

**RESULTS**

**Data by Length of Cardiopulmonary Bypass Time**

In comparing patients with CPB times of less than 240 minutes (N=1,793, 84%) to those with CPB times of 240 minutes or more (N=336, 16%), several significant differences were observed (Supplemental Table 1). The group with longer CPB times had a lower percentage of males (67% vs. 75%, p = 0.003) and a higher incidence of associated CHD at initial diagnosis (37% vs. 17%, p < 0.001). Redo surgeries were more frequent in the longer CPB group (31% vs. 12%, p < 0.001), as were failed initial procedures (6.3% vs. 1.5%, p < 0.001).

The Ross procedure and annulus enlargement were more common in the group with longer CPB times (32% vs. 13%, p < 0.001 for Ross; 16% vs. 4.2%, p < 0.001 for annulus enlargement). Additionally, associated cardiac procedures were more frequently performed in this group (54% vs. 32%, p < 0.001).

Regarding hospitalization, patients with CPB times ≥240 minutes had longer stays in the ICU (median 3 days, IQR 2, 6 vs. 2 days, IQR 1, 3, p < 0.001) and overall hospital stay (median 11 days, IQR 7, 19 vs. 8 days, IQR 6, 12, p < 0.001). Major postoperative complications were significantly higher in the longer CPB group (25% vs. 13%, p < 0.001). However, reoperations on the aortic valve within the same hospitalization were similar between groups (1.5% vs. 1.6%, p = 0.86). Operative mortality was significantly higher in the group with longer CPB (5.4% vs. 0.7%, p < 0.001).

**Data By Mechanism of Aortic Valve Disease**

When comparing patients with aortic valve regurgitation (N=948, 45%), stenosis (N=520, 24%), and mixed disease (N=661, 31%), several significant differences were observed (Figure 1) (Supplemental Table 2). Patients with mixed disease were slightly older at the time of surgery, with a median age of 15 years (IQR 13, 16) compared to 14 years in the regurgitation (IQR 12, 17) and stenosis groups (IQR 12, 16) (p = 0.01). There was no significant difference in male gender distribution, weight, height, or BSA across the three groups.

Associated CHD at initial diagnosis were more common in the regurgitation group (31%) compared to the stenosis (13%) and mixed (9%) groups (p < 0.001). Similarly, VSD, D-TGA, and truncus arteriosus were more frequently associated with regurgitation (p < 0.001). Regurgitation patients also had a higher prevalence of genetic diseases (p < 0.001) and aortic valve endocarditis (p < 0.001). Rheumatic aortic valve disease was significantly more common in the regurgitation group (16%) compared to the stenosis (1%) and mixed disease groups (2%) (p < 0.001).

Regarding the index procedure, aortic valve replacement was more common in the regurgitation group (58%) compared to the stenosis (33%) and mixed groups (48%, p < 0.001), while the Ross procedure was more frequent in patients with stenosis (22%) and mixed disease (26%) (p < 0.001) (Figure 1). Associated cardiac procedures were also more common in the regurgitation group (47%) than in the stenosis (27%) and mixed (26%) groups (p < 0.001).

Hospitalization outcomes showed that patients with regurgitation had a slightly longer stay (p = 0.03). Reoperations on the aortic valve within the same hospitalization were not significantly different between groups. Major postoperative complications were more common in the regurgitation group (19%) compared to stenosis (15%) and mixed (8.9%), but not significant (p = 0.26). Operative mortality was higher in the stenosis (2.1%) and regurgitation (1.9%) groups compared to mixed disease (0.3%, p = 0.01).

**Data by Body Surface Area**

In this analysis, patients were grouped based on their BSA, based on the 25^th^ and 75^th^ percentiles of the overall population. Therefore, the groups included subjects with a BSA less than 1.3 (N=413, 21%), with a BSA between 1.3 and 1.8 (N=1143, 57%), and with a BSA greater than 1.8 (N=453, 21%) (Figure 3) (Supplemental Table 3).

The median age at the time of index surgery was significantly lower in the BSA < 1.3 group (12 years, IQR 11, 13) compared to 15 years (IQR 13, 16) in the 1.3 ≤ BSA ≤ 1.8 group, and 16 years (IQR 15, 18) in the BSA > 1.8 group (p < 0.001). Similarly, weight and height increased with larger BSA, with median weights of 33 kg (IQR 29, 37), 53 kg (IQR 46, 59), and 78 kg (IQR 70, 88), and heights of 142 cm (IQR 137, 148), 162 cm (IQR 156, 170), and 178 cm (IQR 172, 183), respectively (p < 0.001). The proportion of male patients was also higher in the larger BSA groups, reaching 89% in the BSA > 1.8 group compared to 69% in the BSA < 1.3 group (p < 0.001).

Associated CHD at initial diagnosis was more common in the BSA < 1.3 group (27%) compared to 19% in the 1.3 ≤ BSA ≤ 1.8 group and 13% in the BSA > 1.8 group (p < 0.001). However, the prevalence of associated genetic disease did not differ significantly across the groups (p = 0.81).

Regarding the mechanism of aortic valve disease, patients with BSA > 1.8 had a higher proportion of aortic valve regurgitation (47%), while those in the 1.3 ≤ BSA ≤ 1.8 group had the highest rate of mixed aortic valve disease (34%) (p = 0.003).

Redo surgery was more common in patients with BSA < 1.3 (20%) compared to 14% in the middle group and 11% in the BSA > 1.8 group (p = 0.001). The type of index procedure also varied significantly between groups (p < 0.001), with aortic valve repair being more common in the BSA < 1.3 group (43%) compared to aortic valve replacement, which was most frequent in the BSA > 1.8 group (59%). The requirement of annulus enlargement decreased in patient with higher BSA both for aortic valve replacement and Ross operation (p=0.01 for both). Associated cardiac procedures were more common in patients with BSA < 1.3 (46%) compared to 35% and 26% in the higher BSA groups (p < 0.001).

Postoperative outcomes, such as ICU discharge and final discharge, were generally similar across groups, though patients with BSA < 1.3 had a slightly longer median ICU stay. Operative mortality was not significantly different between groups, with rates of 2.4% in the BSA < 1.3 group, 1.0% in the middle group, and 1.8% in the BSA > 1.8 group (p = 0.14).

**SUPPLEMENTAL FIGURES**

**Supplemental Figure 1. Consort Diagram of The Study**


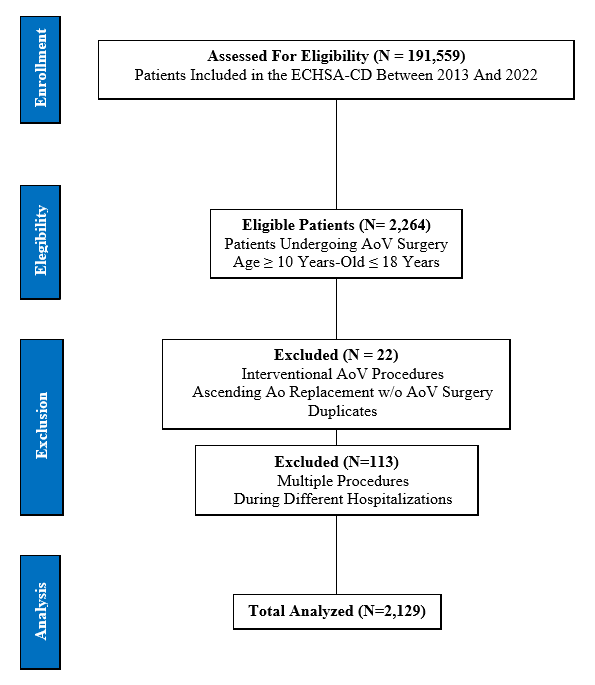


**Supplemental Figure 2. ROC curve showing area under the curve, sensitivity and specificity for the cardiopulmonary bypass time in association with operative mortality.
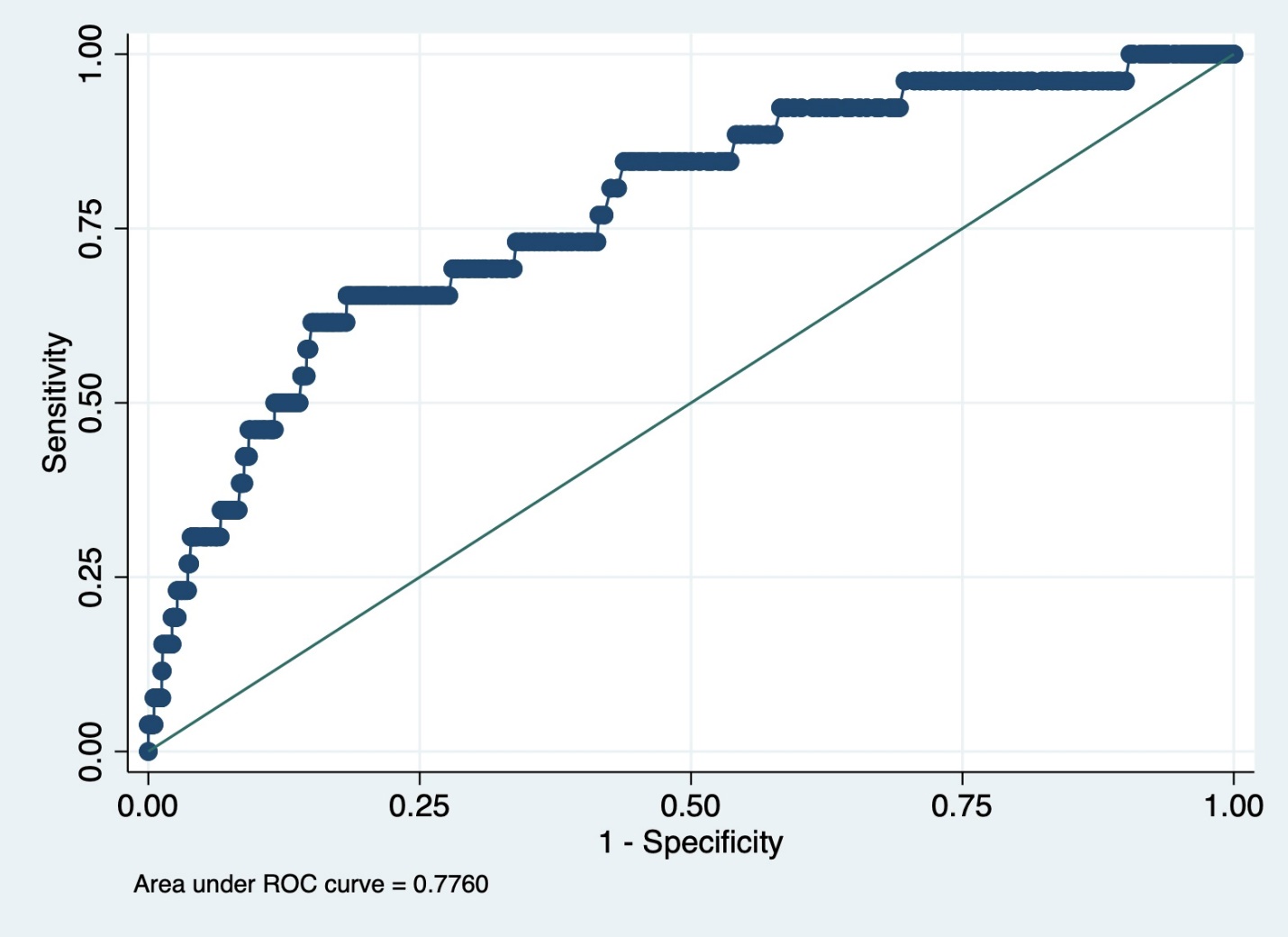
**

**SUPPLEMENTAL TABLES**

| **Supplemental Table 1. Predictors for Mortality** | | |
| --- | --- | --- |
| **Univariate Analysis** | | |
| **Variable** | **OR (95% CI)** | **p-value** |
| Age At Index Surgery | 1.0 (0.9, 1.2) | 0.99 |
| Male Gender | 0.9 (0.4, 1.9) | 0.72 |
| Weight (Kg) | 0.9 (1.0, 1.1) | 0.51 |
| BSA (m2) | 0.5 (0.1, 1.5) | 0.19 |
| Associated CHD at Initial Diagnosis | 4.5 (2.2, 9.1) | **<0.001** |
| Associated Genetic Disease | 1.4 (0.3, 5.9) | 0.66 |
| Mechanism Of Aortic Valve Disease | 0.6 (0.3, 0.9) | **0.022** |
| Period Of Surgery | 0.9 (0.5, 1.9) | 0.84 |
| Redo Surgery | 7.2 (3.5, 15) | **<0.001** |
| Failed Initial Index Procedure | 3.1 (0.7, 14) | 0.13 |
| Type Of Index Procedure | 1.4 (0.8, 2.3) | 0.20 |
| Annulus Enlargement | 3.8 (1.5, 9.5) | **0.004** |
| Associated Cardiac Procedure | 1.5 (0.7, 3.1) | 0.26 |
| CPB Time | 1.1 (1.1, 1.2) | **<0.001** |
| AOX Time | 1.1 (1.1, 1.2) | **0.008** |
| Circulatory Arrest | 6.5 (1.9, 22) | **0.003** |
| CHD: Congenital Heart Disease, CPB: Cardiopulmonary Bypass, AOX: Aortic Cross-Clamp. Variables with P<0.05 on univariable analysis were included in the multivariable model. | | |

| **Supplemental Table 2. Data by Length of Cardiopulmonary Bypass Time (N, % or median, IQR).** | | | |
| --- | --- | --- | --- |
|  | **CPB < 240 min**  **(N=1793, 84%)** | **CPB ≥ 240 min**  **(N=336, 16%)** | **p-value** |
| **Demographics** |  |  |  |
| Age At Index Surgery (Years) | 14 (12, 16) | 14 (12, 16) | 0.56 |
| Male Gender | 1344 (75%) | 226 (67%) | **0.003** |
| Weight (Kg) | 53 (42, 66) | 52 (40, 65) | 0.21 |
| Height (Cm) | 162 (151, 173) | 162 (152, 170) | 0.59 |
| BSA (m2) | 1.6 (1.3, 1.8) | 1.5 (1.3, 1.8) | 0.33 |
| **Associated Disease** |  |  |  |
| Associated CHD At Initial Diagnosis | 296 (17%) | 124 (37%) | **<0.001** |
| Associated Genetic Disease | 82 (4.6%) | 20 (6.0%) | 0.28 |
| **Preoperative Data** |  |  |  |
| Mechanism Of Aortic Valve Disease |  |  | 0.098 |
| Aortic Valve Regurgitation | 784 (44%) | 164 (49%) |  |
| Aortic Valve Stenosis | 436 (24%) | 84 (25%) |  |
| Mixed Aortic Valve Disease | 573 (32%) | 88 (26%) |  |
| Endocarditis On the Aortic Valve | 67 (3.7%) | 17 (5.1%) | 0.25 |
| **Index Procedure** |  |  |  |
| Period Of Surgery |  |  | 0.53 |
| 2013-2017 | 1015 (57%) | 184 (55%) |  |
| 2018-2022 | 778 (43%) | 152 (45%) |  |
| Redo Surgery | 214 (12%) | 105 (31%) | **<0.001** |
| Failed Initial Index Procedure | 26 (1.5%) | 21 (6.3%) | **<0.001** |
| Type Of Index Procedure |  |  | **<0.001** |
| Aortic Valve Repair | 699 (39%) | 43 (13%) |  |
| Aortic Valve Replacement | 859 (48%) | 185 (55%) |  |
| Ross Procedure | 235 (13%) | 108 (32%) |  |
| Annulus Enlargement | 75 (4.2%) | 55 (16%) | **<0.001** |
| **Associated Procedure** |  |  |  |
| Associated Cardiac Procedure | 575 (32%) | 182 (54%) | **<0.001** |
| **Hospitalization** |  |  |  |
| POD At ICU Discharge | 2 (1, 3) | 3 (2, 6) | **<0.001** |
| POD At Final Discharge | 8 (6, 12) | 11 (7, 19) | **<0.001** |
| **Reoperation Within Same Hospitalization** |  |  |  |
| Reoperation On the Aortic Valve | 29 (1.6%) | 5 (1.5%) | 0.86 |
| **Postoperative Complication** |  |  |  |
| Major Complications Requiring Treatment | 234 (13%) | 85 (25%) | **<0.001** |
| **Mortality** |  |  |  |
| Operative Mortality | 13 (0.7%) | 18 (5.4%) | **<0.001** |
| CHD: Congenital Heart Disease, POD: Postoperative Day. | | | |

| **Supplemental Table 3. Data By Mechanism of Aortic Valve Disease (N, % or median, IQR).** | | | | |
| --- | --- | --- | --- | --- |
|  | **Regurgitation**  **(N=948, 45%)** | **Stenosis**  **(N=520, 24%)** | **Mixed**  **(N=661, 31%)** | **p-value** |
| **Demographics** |  |  |  |  |
| Age At Index Surgery (Years) | 14 (12, 17) | 14 (12, 16) | 15 (13, 16) | **0.012** |
| Male Gender | 682 (72%) | 397 (76%) | 491 (74%) | 0.17 |
| Weight (Kg) | 52 (41, 67) | 52 (41, 65) | 55 (44, 66) | 0.18 |
| Height (Cm) | 162 (151, 173) | 160, 149, 171) | 164 (154, 173) | 0.19 |
| BSA (m2) | 1.5 (1.3, 1.8) | 1.5 (1.3, 1.8) | 1.6 (1.4, 1.8) | 0.14 |
| **Associated Disease** |  |  |  |  |
| Associated CHD At Initial Diagnosis | 292 (31%) | 68 (13%) | 57 (9%) | **<0.001** |
| VSD (Any Form) | 91 (9.6%) | 13 (2.5%) | 16 (2.4%) | **<0.001** |
| D-TGA | 58 (6.1%) | 2 (0.4%) | 1 (0.2%) | **<0.001** |
| Truncus Arteriosus | 38 (4.0%) | 0 (0%) | 2 (0.3%) | **<0.001** |
| Associated Genetic Disease | 65 (6.9%) | 19 (3.7%) | 18 (2.7%) | **<0.001** |
| **Preoperative Data** |  |  |  |  |
| Endocarditis On the Aortic Valve | 59 (6.2%) | 6 (1.2%) | 19 (2.9%) | **<0.001** |
| Rheumatic Aortic Valve Disease | 151 (16%) | 5 (1.0%) | 13 (2.0%) | **<0.001** |
| **Index Procedure** |  |  |  |  |
| Type Of Index Procedure |  |  |  | **<0.001** |
| Aortic Valve Repair | 343 (36%) | 231 (44%) | 168 (25%) |  |
| Aortic Valve Replacement | 552 (58%) | 174 (33%) | 318 (48%) |  |
| Ross Procedure | 53 (6%) | 115 (22%) | 175 (26%) |  |
| **Associated Procedure** |  |  |  |  |
| Associated Cardiac Procedure | 445 (47%) | 141 (27%) | 171 (26%) | **<0.001** |
| **Hospitalization** |  |  |  |  |
| POD At ICU Discharge | 2 (1, 3) | 2 (1, 3) | 2 (1, 3) | 0.30 |
| POD At Final Discharge | 8 (7, 14) | 8 (6, 12) | 8 (6, 11) | **0.032** |
| **Reoperation Within Same Hospitalization** |  |  |  |  |
| Reoperation On the Aortic Valve | 18 (1.9%) | 6 (1.2%) | 10 (1.5%) | 0.68 |
| **Postoperative Complication** |  |  |  |  |
| Major Complications Requiring Treatment | 182 (19%) | 78 (15%) | 59 (8.9%) | 0.26 |
| **Mortality** |  |  |  |  |
| Operative Mortality | 18 (1.9%) | 11 (2.1%) | 2 (0.3%) | **0.010** |

CHD: Congenital Heart Disease, D-TGA: D-Transposition of The Great Arteries, POD: Postoperative Day, ICU: Intensive Care Unit.

| **Supplemental Table 4. Data By Body Surface Area (N, % or median, IQR).** | | | | |
| --- | --- | --- | --- | --- |
|  | **BSA<1.3**  **(N=413, 21%)** | **1.3≤BSA≤1.8**  **(N=1143, 57%)** | **BSA>1.8**  **(N=453, 21%)** | **p-value** |
| **Demographics** |  |  |  |  |
| Age At Index Surgery (Years) | 12 (11, 13) | 15 (13, 16) | 16 (15, 18) | **<0.001** |
| Male Gender | 283 (69%) | 808 (71%) | 405 (89%) |  |
| Weight (Kg) | 33 (29, 37) | 53 (46, 59) | 78 (70, 88) | **<0.001** |
| Height (Cm) | 142 (137, 148) | 162 (156, 170) | 178 (172, 183) | **<0.001** |
| BSA (m2) | 1.2 (1.1, 1.2) | 1.6 (1.4, 1.7) | 1.9 (1.9, 2.1) | - |
| **Associated Disease** |  |  |  |  |
| Associated CHD At Initial Diagnosis | 111 (27%) | 216 (19%) | 57 (13%) | **<0.001** |
| Associated Genetic Disease | 18 (4.4%) | 53 (4.6%) | 19 (4.2%) | 0.81 |
| **Preoperative Data** |  |  |  |  |
| Mechanism Of Aortic Valve Disease |  |  |  | **0.003** |
| Aortic Valve Regurgitation | 189 (46%) | 486 (43%) | 215 (47%) |  |
| Aortic Valve Stenosis | 121 (29%) | 263 (23%) | 100 (22%) |  |
| Mixed Aortic Valve Disease | 103 (25%) | 394 (34%) | 138 (30%) |  |
| **Index Procedure** |  |  |  |  |
| Period Of Surgery |  |  |  | 0.58 |
| 2013-2017 | 234 (57%) | 628 (55%) | 260 (57%) |  |
| 2018-2022 | 179 (43%) | 515 (45%) | 193 (43%) |  |
| Redo Surgery | 81 (20%) | 157 (14%) | 51 (11%) | **0.001** |
| Failed Initial Index Procedure | 10 (2.4%) | 25 (2.2%) | 8 (1.8%) |  |
| Type Of Index Procedure |  |  |  | **<0.001** |
| Aortic Valve Repair | 179 (43%) | 405 (35%) | 116 (26%) |  |
| Aortic Valve Replacement | 167 (40%) | 558 (49%) | 269 (59%) |  |
| Annulus enlargement | 14 (3.4%) | 33 (2.9%) | 9 (2.0%) | **0.012** |
| Ross Procedure | 67 (16%) | 180 (16%) | 68 (15%) |  |
| Annulus enlargement | 18 (4.4%) | 35 (3.0%) | 7 (1.5%) | **0.011** |
| **Associated Procedure** |  |  |  |  |
| Associated Cardiac Procedure | 189 (46%) | 395 (35%) | 119 (26%) | **<0.001** |
| **Reoperation Within Same Hospitalization** |  |  |  |  |
| Reoperation On the Aortic Valve | 6 (1.5%) | 21 (1.8%) | 5 (1.1%) | 0.48 |
| **Postoperative Complication** |  |  |  |  |
| Major Complication Requiring Treatment | 69 (17%) | 168 (15%) | 68 (15%) | 0.62 |
| **Hospitalization** |  |  |  |  |
| POD At ICU Discharge | 2 (1, 4) | 2 (1, 3) | 2 (1, 3) | **0.022** |
| POD At Final Discharge | 9 (7, 14) | 8 (6, 13) | 8 (6, 12) | 0.34 |
| **Mortality** |  |  |  |  |
| Operative Mortality | 10 (2.4) | 12 (1.0%) | 8 (1.8%) | 0.14 |

BSA: Body Surface Area, CHD: Congenital Heart Disease, D-TGA: D-Transposition of The Great Arteries, POD: Postoperative Day, ICU: Intensive Care Unit.
